# Mechanisms by Which Traditional Chinese Medicines Influence the Intestinal Flora and Intestinal Barrier

**DOI:** 10.3389/fcimb.2022.863779

**Published:** 2022-04-28

**Authors:** Qingya Che, Tingting Luo, Junhua Shi, Yihuai He, De-Lin Xu

**Affiliations:** ^1^ Department of Medical Cell Biology, Zunyi Medical University, Zunyi, China; ^2^ Department of Infectious Diseases, The Affiliated Hospital of Zunyi Medical University, Zunyi, China

**Keywords:** traditional Chinese medicine, intestinal flora, intestinal mucosal barrier, effect mechanism, review

## Abstract

The effect of a drug on the intestinal flora and the intestinal barrier is an important evaluation index for drug safety and efficacy. Chemical synthetic drugs are widely used due to their advantages of fast efficacy and low doses, but they are prone to cause drug resistance and inhibit proton pumps, which may harm intestinal health. Traditional Chinese medicine (TCM) has been applied clinically for thousands of years, and how TCMs regulate intestinal health to achieve their effects of disease treatment has become a hot research topic that needs to be resolved. This paper reviews the recent research on the effects of TCMs on intestinal microorganisms and the intestinal mucosal barrier after entering the intestine, discusses the interaction mechanisms between TCMs and intestinal flora, and details the repair effect of TCMs on the intestinal mucosal barrier to provide a reference for the development, utilization, and modernization of TCM.

## Introduction

**Figure 1:**
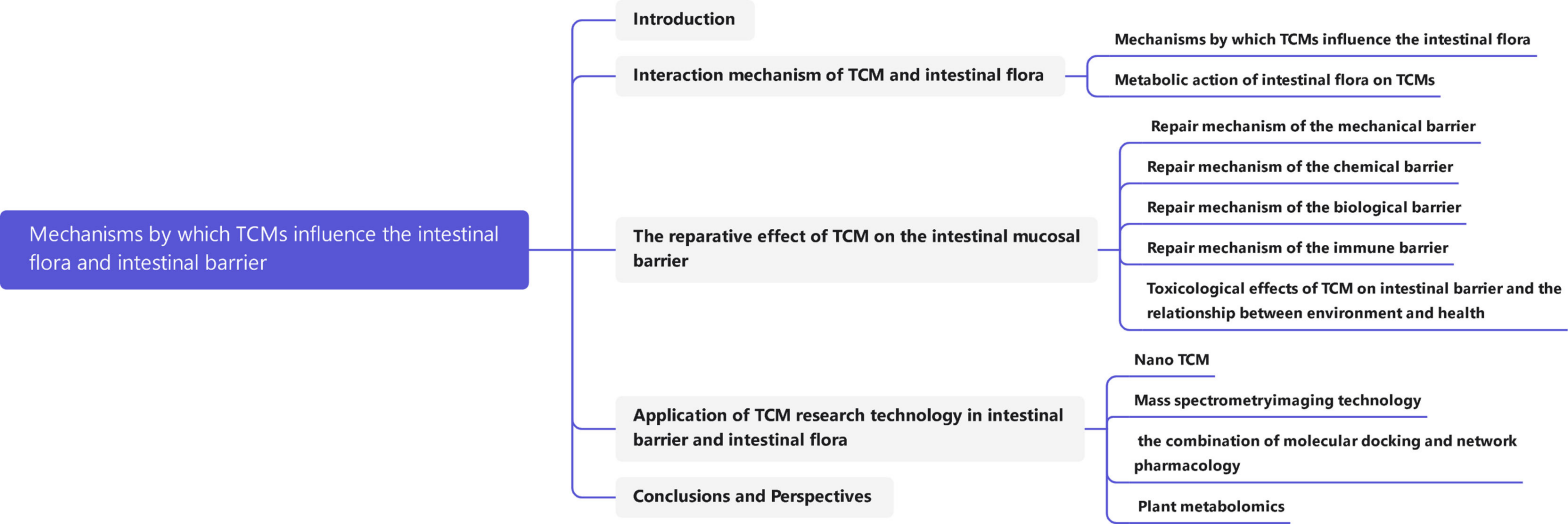
Article Mind Map.

In recent years, with the rise in public attention to intestinal health, the intestine has become a research hotspot in the field of traditional Chinese medicines (TCMs). The intestinal flora and intestinal barrier are likely to be important targets through which most Chinese medicines exert their effects and treat diseases. Studies have shown that the balance of intestinal flora and the stability of the intestinal barrier system are the basis for the physiological functions of the intestinal tract. Intestinal flora is a general term for the bacteria living in the human intestine. They help maintain a good environment in the intestine, and their physiological function has become an indispensable part of the physiological function of the host, playing an active role in material anabolism, catabolism, etc (Valdes et al., 2018). The intestinal barrier system refers to a functional isolation belt that prevents harmful substances in the intestinal lumen from entering the blood circulation, to maintain the relative stability of the body’s internal environment and maintain the normal life activities of the body (Duan et al., 2019). In the process of evolution, the intestine and its microbiota have come to complement each other and jointly maintain the health of the body, but when they are stressed by factors from the environment, diet, or drugs, their function will be seriously affected, thereby inducing intestinal metabolic disorders, raising the chances of pathogens invading the body, and increasing the risk of diseases such as diabetes, obesity, and metabolic syndrome (Lin et al., 2021). At present, chemical synthetic drugs dominate in the treatment of diseases caused by intestinal problems, but their antibiotics, proton pump inhibitors and other components also subtly endanger human intestinal health in the process of treating diseases. TCMs, with the advantages of mild antibacterial activity, reparative action, and the fact that humans do not easily develop resistance to them, have gotten much attention for the possibility of microflora balance regulation and intestinal barrier repair. However, due to the complexity and diversity of the active ingredients of TCMs, their interactions with the intestinal flora and their repair mechanisms of the intestinal barrier are not fully clear. This paper summarizes the mechanisms by which TCMs influence the intestinal flora and the intestinal barrier system as discovered in recent years to provide a reference for the development, utilization, and modernization of TCM. (The article map is shown in **Figure 1**)

## Interaction Mechanism of TCM and Intestinal Flora

The interaction between TCMs and intestinal flora is bidirectional: on the one hand, TCMs can regulate the structure and metabolic function of the flora by selectively inhibiting or promoting the growth of different types of intestinal microorganisms, thereby promoting human health. On the other hand, the intestinal flora will metabolize TCMs, which may increase efficacy or reduce toxicity, or may generate toxic metabolites.

### Mechanisms by Which TCMs Influence the Intestinal Flora

**Figure 2:**
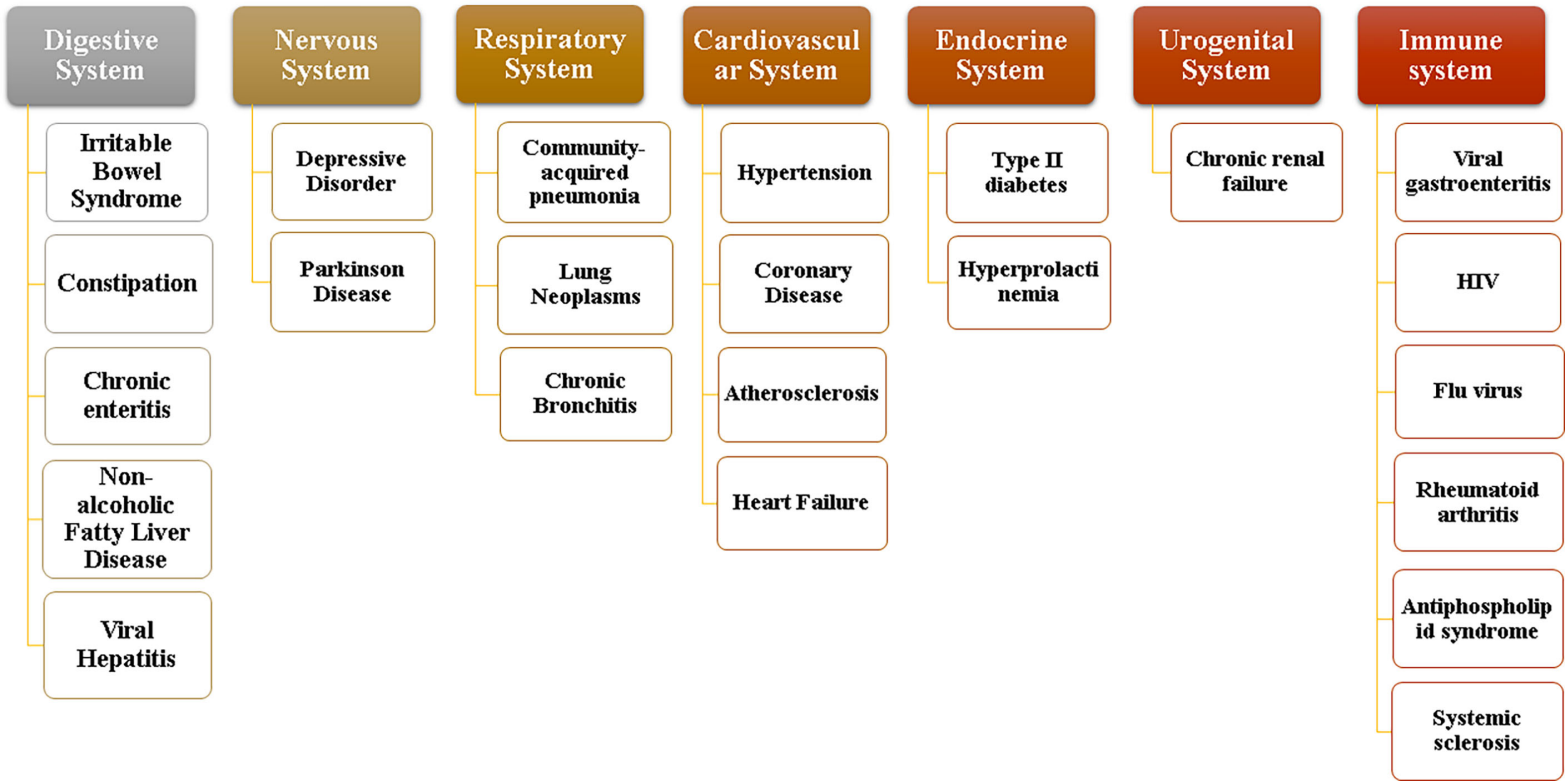
System diseases involving intestinal flora.

Normal flora mainly plays a positive physiological role in the gastrointestinal tract in the following four aspects. (1) Maintain the stability of the intestinal environment: the normal flora has the physical barrier function to resist the invasion of foreign bacteria and prevent the translocation of opportunistic pathogenic bacteria, and can also provide some nutrients for intestinal mucosal cells (Pushpanathan et al., 2019). (2) Involved in substance metabolism: Intestinal microflora contains more metabolic enzymes than the host genome, so it has more powerful metabolic functions. Its metabolites, such as short-chain fatty acids, secondary bile acids, sulfides and indoles, are key factors for improving the progress and prognosis of diseases based on intestinal flora (Sittipo et al., 2019). (3) Enhance immune function: the intestine is one of the organs with the most immune cells in the human body. Intestinal bacteria and their metabolites can regulate the immune system through various ways to promote the maturity of immune cells and the normal development of immune function (Belkaid and Harrison, 2017). (4) Affect brain function and behavior: gut microbiota can be associated with the central nervous system, affecting brain function and host behavior by regulating anxiety, emotion, cognition and pain (Xu et al., 2020). When the environment, diet, drugs and other factors cause the flora imbalance, the above functions may be affected, and severe cases may cause various system diseases as shown in **Figure 2**. According to the relevant data, dysbacteriosis can lead to significant changes in the expression of functional genes of flora and the activity of metabolic related enzymes, thereby affecting their normal physiological functions (Nogacka et al., 2019). At the same time, it can also cause the destruction of intestinal barrier, leading to the occurrence of toxic metabolites into blood and bacterial translocation, resulting in the body’s inflammatory reaction to occur or worsen. In addition, the number and function of regulatory T cells are also hindered by intestinal flora disturbance, resulting in weakened response regulation of T cells to Th1, Th2 and Th17 effector cells, affecting the normal immune function of the body (Matsuoka and Kanai, 2015). These changes will have a serious impact on host health.

Clinical aseptic models or fecal transplantation have proved that intestinal flora can be used as targeted bacteria to achieve the purpose of TCM treatment of diseases, mainly reflected in the influence of the active ingredients of TCMs on the structure, composition and metabolites of intestinal flora. For example, Wuyao extract can regulate intestinal flora disturbance and affect bile acid metabolism, thereby improving hyperlipidemia (Jiang et al., 2021). Through PCR-DGGE and LC-MS detection, it was found that the number of *Bifidobacterium* and *Lactobacillus* and the content of short-chain fatty acids in the cecum of the constipation model rats treated with hemp seed oil increased, while the proportion of harmful bacteria decreased significantly, which effectively improved the constipation symptoms of rats (Hanbing et al., 2018). Other TCMs also showed good therapeutic effects on improving corresponding diseases based on intestinal flora and its metabolites, as shown in **Table 1**. At the same time, relevant studies have shown that the interaction between TCM and TCM can achieve the effect of reducing toxicity, for example, *Aconitum carmichaeli Debx* has great toxicity, often combined with *Radix ginseng* for the treatment of cardiovascular system, respiratory system and nervous system diseases. Compared with *Aconitum carmichaeli Debx* alone, the combination of *Aconitum carmichaeli Debx* and *Radix ginseng* can improve the intestinal flora of normal rats and promote the proliferation of beneficial bacteria, and when the ratio of the two was 1:2, the content of *Lactobacillus* in the intestinal tract was higher than that of the *Aconitum carmichaeli Debx* alone and the ratio of 1:1 compatibility group (Tang et al., 2018). But when the side effect of TCM is strong or the combination of TCM is wrong, it may not achieve the therapeutic effect or even aggravate the disease. For example, Yuanhuapin is both as active and toxic ingredient in *Genkwa flos*. It can cause intestinal flora disorder in normal rats, resulting in significant changes in the contents of phenylacetylglycine, maleic acid and 3-ethyldioxyindole, the metabolites of intestinal flora, thereby affecting amino acid metabolism, lipid and glucose metabolism and other metabolic pathways, leading to toxic reactions in intestine and liver (Chen et al., 2016). Qianjinzi is similar to *Euphorbia* in “the eighteen incompatible medicaments” in terms of efficacy, basis and chemical composition. Using it in combination with *Glycyrrhiza uralensis Fisch* increased the number of harmful bacteria such as *Enterococcus* and S24_7_ukn, and enhanced the metabolic capacity of intestinal flora at the same time, which increases the content of toxic substances such as indole and p-cresol, thereby aggravating the intestinal injury in mice. (Tao et al., 2018).

**Table 1:** Regulation of intestinal flora and its metabolites by TCMs.

TCM	Animal models	Effect on intestinal flora abundance	Effects on gut microbiota metabolites	Therapeutic effect	Mechanism of action	Document
Barley leaf	Colitic mice	*Proteobacteria*↑*Enterobacteriaceae*↓	Inosine, Guanosine↑	Reduces the severity of disease and microbial imbalance	Adenosine is produced by gut microbes to activate PPARγ signalling	(Li et al., 2021)
Gegen cenlian decoction	Type 2 diabetic rat	*Faecalibacterium, Roseburia*↑	SCFAs↑	Reduces systemic and local inflammation in rats	Increases the content of butyric acid	(Xu et al., 2020)
*Ginseng*	Obese mice	*E.faecalis*↑	Nutmeg oleic acid↑	Antiobesity action	Activates BAT and form brown fat to increase energy metabolism	(Quan et al., 2020)
Pu-erh tea	Hyperlipidaemia mice	*Lactobacillus, Bacillus, Enterococcus, Lactococcus, Streptococcus*↓	Cholesterol, Fat↓	Decreases liver and serum cholesterol levels	Inhibits microorganisms associated with bile salt hydrolase activity and increases ileal binding bile acid levels	(Huang et al., 2019)
*Rhubarb*	Ulcerative colitis mice	*Lactobacillus*↑	Uric acid↓	Alleviates dextran sulfate sodium-induced ulcerative colitis	Reduces the concentration of uric acid, the end product of intestinal purine metabolism	(Wu et al., 2020)
Luteolin	Ulcerative colitis mice	*Lactobacillus/Prevotella_9*↓	Amino acids, Starch, Sucrose↑	Colonic injury significantly reduced and inflammation effectively improved	Inhibition of inflammatory factors expression	(Li et al., 2021)
*Radix Paeoniae Alba*	Autoimmune thyroiditis rats	*Lactobacillus, Prevotellaceae, Romboutsia*↑ *Firmicutes*↓	SCFAs↑	Adjusts the composition and diversity of intestinal flora and improves intestinal mucosal injury	Regulation of inflammatory factors and sIgA to alleviate thyroid follicular injury and colonic mucosal lesion	(Mu et al., 2021)
GeGen QinLian decoction	Influenza virus infectious mice	*Akkermansia_muciniphila, Desulfovibrio_C21_c20, Lactobacillus_salivarius*↑ *Escherichia_coli*↓	—	Effectively protecting mice from influenza virus-infected pneumonia	affect systemic immunity, at least in part, through the intestinal flora, thereby protect the mice against influenza virus infectious pneumonia	(Deng et al., 2021)
Wenyang Jiedu Huayu prescription	HBV related liver failure	*bifidobacterium*↑ *enterobacteria*↓	Endotoxin↓	Effectively reduce endotoxemia and improve clinical efficacy	Reversing intestinal flora imbalance	(Wen-Fang et al., 2014)
*Ephedra sinica*	H1N1 virus infected mice	*Lactobacillales, Bifidobacteriaceae*↑	SCFAs↑	Significantly treats acute lung injury caused by H1N1	Regulate the type of bacteria and metabolites and inhibit the release of inflammatory factors	(Xiaoting et al., 2020)

↓ refers to decline, ↑ refers to increase, — refers to no effect.

### Metabolic Action of Intestinal Flora on TCMs

Scientific research has shown that the intestinal microbiome encodes about 3.3 million genes, far more than humans. They have many enzymes that the human body does not have, and play an important role in the metabolism and transformation of TCM (Qin et al., 2010). Relevant studies have proved that intestinal flora can produce low polarity and relatively stable molecular mass of TCM metabolites through hydrolysis, oxidation, reduction and isomerization reactions, which can accelerate the intestinal absorption and improve the bioavailability of TCM (Xu et al., 2017). For example, Most glycosides in Huangqin Decoction can be digested and absorbed by the body through the catalytic deglycosylation of intestinal flora (Zuo et al., 2002). The ginsenosides can be transformed into hydrophobic compounds under the combined action of gastric juice and intestinal microorganisms, such as protopanaxadiol-type ginsenosides are mainly converted into compounds K and ginsenoside Rh2. Compared with protopanaxadiol-type ginsenosides, the transformed metabolite compound K exhibits more effective pharmacological effects such as antitumor, anti-inflammatory, antidiabetic, antiallergic and neuroprotective (Kim et al., 2018). At the same time, intestinal flora also has the effect of reducing toxicity to TCMs. For example, *Aconitum carmichaeli Debx* is widely used in clinical practice, but it must be used with caution because of its high toxicity. According to reports, in addition to processing and compatibility to achieve attenuation, it can also achieve attenuation through intestinal flora metabolism.

On the other hand, the side effects of intestinal flora on TCM are also worthy of further study. In recent years, many cases of bitter amygdala poisoning have been also reported. For example, the normal rats receiving 600 mg/kg amygdalin showed symptoms of drowsiness, convulsions, and death within 2 to 5 hours, while sterile rats did not show obvious symptoms of poisoning at the same dose, when both groups were received at a non-toxic oral dose of 50 mg/kg, the recovery rate of amygdalin in normal rats was lower than that of sterile rats, and only amygdalin was detected in sterile rat feces (Carter et al., 1980). Further studies have shown that oral amygdinoside was hydrolyzed by β-glucosidase of the intestinal flora to produce the toxic substance hydrocyanic acid, which triggers a serious toxic reaction (Liu et al., 2017). Of course, the intestinal flora also has an improving effect on the toxic side effects of TCMs. For example, aconitine Chinese medicines such as Chuanwu and Caowu have been confirmed that they can be converted into mono- and di-lipids with lower toxicity under the acylation and esterification of intestinal microorganisms (Zhao et al., 2008).

**Figure 3:**
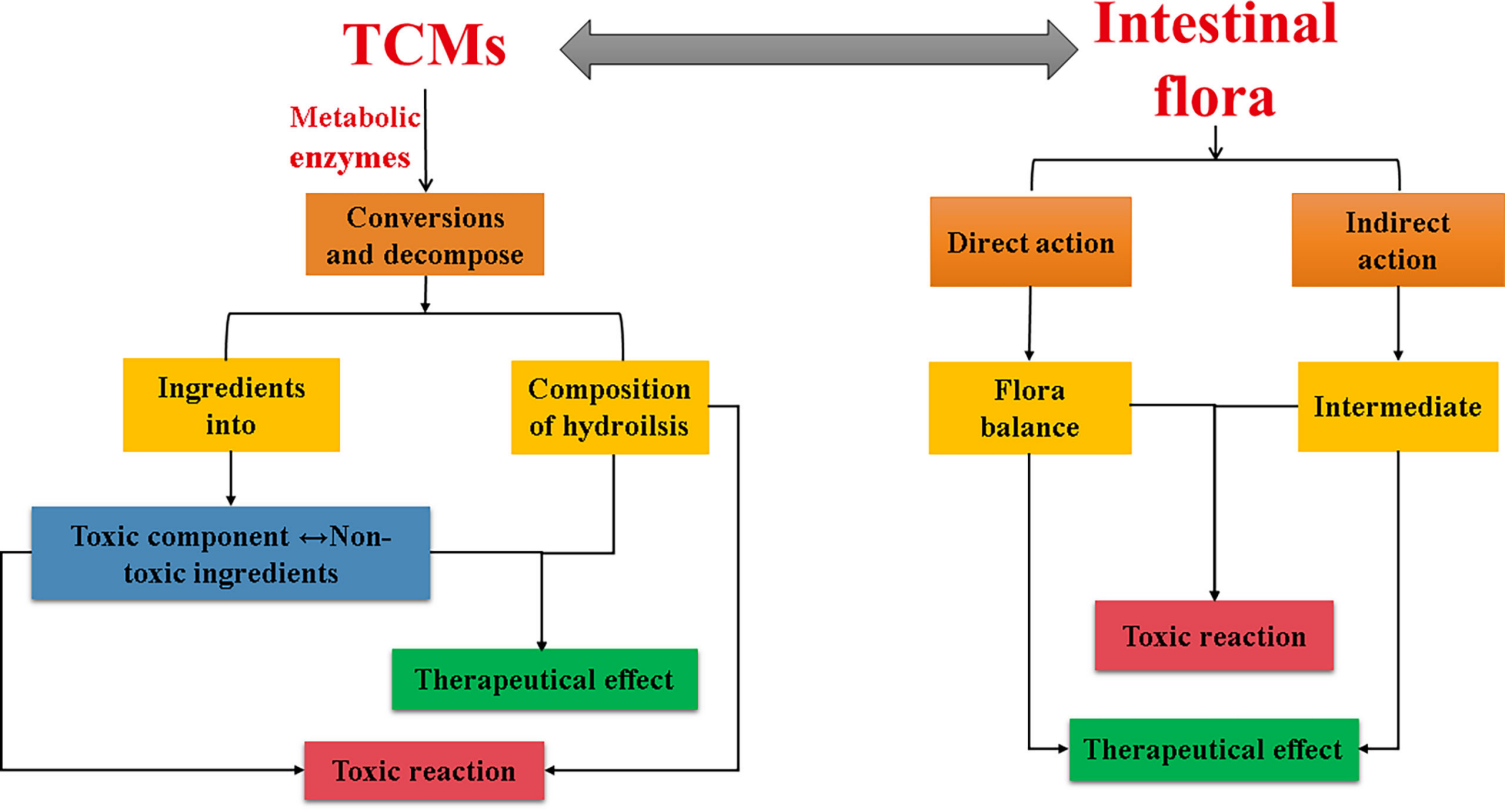
Interaction between TCM and intestinal flora.

To sum up, the interaction between TCMs and intestinal flora is both positive and negative, and the two complement each other and are closely related (**Figure 3**), showing great potential in the treatment and prevention of diseases. In addition, the recent application of TCMs in the treatment of novel coronavirus and influenza viruses has achieved good efficacy. Some studies have reported that the treatment of virus-related diseases with TCM may be related to the intestinal microecology, such as Houttuynia cordata can reverse the composition change of intestinal microbiota caused by H1N1 infection, with significantly reduced relative abundances of Vibrio and Bacillus, the pathogenic bacterial genera (Chen et al., 2019). The latest researches have shown that the gut microbiota plays an important role in the progression of COVID-19, and that gut microbiota imbalance and endotoxemia may accelerate the progression of COVID-19 (Gou et al., 2020). Many TCMs can support virus-affected body organs and systems, restore the structure of the gut flora, and exhibit properties relevant to COVID-19 treatment (Chen et al., 2021). In-depth exploration of the interaction mechanism between TCM and intestinal flora may provide new research directions and therapeutic targets for diseases including the COVID-19.

## The Reparative Effect of TCM on the Intestinal Mucosal Barrier

The intestinal mucosal barrier is a highly selective functional barrier system present in the intestine (Kraehenbuhl et al., 1997) that can prevent harmful substances such as pathogenic microorganisms and endotoxins from passing through the intestinal mucosa while selectively absorbing nutrients in the intestine. It plays an important role in immune defence and maintaining intestinal mucosal integrity. Under normal conditions, the intestinal mucosal barrier can be divided into a mechanical barrier, biological barrier, chemical barrier, and immune barrier, which jointly exert the function of the intestinal mucosal barrier. TCM can repair the intestinal mucosal barrier in many ways to protect barrier function.

### Repair Mechanism of the Mechanical Barrier by TCM

The mechanical barrier, also known as a physical barrier, is the front-line guard that prevents harmful substances penetrating the intestinal mucosa and is the most important of the intestinal mucosal barriers. Normally, the mechanical barrier is mainly composed of a tight connection between the intestinal mucosal epithelium and cells. When the blood supply of the intestinal mucosa is insufficient, that is, a microcirculation disorder of the intestinal mucosa occurs, the integrity of the intestinal mucosa will be harmed, increasing the permeability of the intestinal mucosa, thereby weakening the selective permeation and barrier function. Many studies have shown that TCMs can maintain the mechanical barrier function by improving the microcirculation of the intestinal mucosa, repairing the integrity of the intestinal mucosa, and reducing the permeability of the intestinal mucosa. Under normal circumstances, the mammalian intestine lacks enzymes that decompose D-lactic acid (D-LA), and the level of diamine oxidase (DAO) in the body is also low; these substances are very rare in the blood. When the mechanical barrier of the intestinal mucosa is broken, the intestinal mucosa will be fully permeable, so they can enter the blood through the intestinal mucosa, and the integrity of the intestinal mucosa can be accurately reflected by measuring these indicators (Le, 2019). The intestinal mucosa also contains tight junction proteins such as occludin, which are important components in the integrity of the intestinal mucosa. Huanglian Jiedu Decoction combined with electroacupuncture can significantly reduce serum DAO levels and serum d-lactic acid levels in critically ill patients undergoing abdominal surgery, thus accelerating the repair of the intestinal mucosal mechanical barrier (Wang et al., 2015). Clinical studies have shown that TNF-α, IL-6 and other inflammatory factors can increase vascular endothelial cell permeability and weaken intestinal mucosal barrier function (Xie et al., 2019). A study also demonstrated that *Bletilla striata* polysaccharide can upregulate the expression of the occludin protein in mice with ulcerative colitis, thereby improving the function and integrity of the epithelial barrier (Li et al., 2021).

### Repair Mechanism of the Chemical Barrier by TCM

The intestinal mucosa chemical barrier is composed of various chemicals, such as mucus, glycoproteins, various digestive enzymes, and lysozymes, which are secreted by epithelial cells of the intestinal mucosa, and bacteriostatic substances, which are secreted by the intestinal flora. These secretions can change the attack site of pathogenic bacteria or opportunistic pathogenic bacteria, affect the colonization ability of bacteria, and mainly play a role in inhibiting bacteria and regulating the intestinal environment (Zhai, 2014). TCM can affect the secretion of mucus and the composition of the mucus layer by regulating the number and secretion capacity of intestinal mucosal epithelial cells and creating a suitable living environment for some intestinal microorganisms while inhibiting others. Study has confirmed that Gegenqinlian Decoction (GQ) regulated the activity of Notch signalling by a bidirectional mechanism, promoted the proliferation and differentiation of goblet cells to accelerate the secretion of viscoelastic gels, and helped complete the repair of the intestinal mucosal epithelium (Zhao et al., 2020). *Aloe vera* significantly up-regulated the expression of mucins (such as MUC2 and MUC5AC) in ulcerative colitis rats, and increased the thickness of mucous layer in colon, thereby accelerating the repair of intestinal mucosa (Shi et al., 2021). In addition, *Blautia* can protect the intestine by producing antibacterial substances and compete for intestinal adhesion sites to inhibit pathogenic bacteria, thereby enhancing intestinal barrier function (Jiang et al., 2018). And ellagic acid can enhance the activities of digestive enzymes such as lactase, sucrase and alkaline phosphatase in jejunum of mice, so as to promote intestinal development and improve antioxidant capacity of mice (Xu et al., 2021). Another study confirmed that *Rhodiola crenulata* can reduce blood endotoxemia by increasing the expression of tight junctions (zonula occlusion-1 and agglutinin) and antimicrobial proteins (Reg3g and lysozyme C) in the small intestine, and improve obesity in mice by regulating the balance of flora (Chang et al., 2018).

### Repair Mechanism of the Biological Barrier by TCM

The intestinal mucosal biological barrier is a layer of the bacterial membrane barrier formed by the attachment of intestinal microorganisms to the intestinal mucosa. It has important value in nutrient absorption, immune defence, and metabolic balance. The intestinal microbiota is a unique and diverse ecosystem, and it is also one of the systems with the highest known cell densities (Lei, 2012). The balance of this system is closely related to obesity, hypertension, and other diseases, and regulating the balance of the microbiota has become a key point in the prevention and control of diseases today. According to relevant studies, external or internal factors are highly likely to affect the attachment sites of certain bacteria on the intestinal surface by altering the glycan structure of the intestinal mucosa, thereby indirectly selectively stimulating the instantaneous growth of certain bacteria to destroy the balance of flora, and eventually lead to the occurrence of intestinal diseases. The repair of biological barriers by TCM is mainly achieved by adjusting the balance of microbial groups or increasing the relative abundance of dominant microbes and the balance between flora and host. Studies have found that the active ingredient in *Pogostemon cablin* can significantly increase the relative abundance of probiotics such as *Lactobacillus* and *Bifidobacterium* and reduce the relative abundance of harmful bacteria such as *Parabacillus*, *Bacteroides*, and *Helicobacter pylori*, thus regulating the balance of the flora and maintaining the intestinal mucosa biological barrier function (Wu et al., 2020). There are many kinds of intestinal microorganisms. Now people only know about the tip of the iceberg, and there are many strains that have not been found. It is an urgent problem to study the metabolic mechanism of intestinal microorganisms in the field of medicine.

### Repair Mechanism of the Immune Barrier by TCM

The main function of the intestine is to realize the digestion and absorption of substances. According to clinical studies, it has more higher concentration of lymphocytes than lymphoid tissues, and they are extremely important immune barriers in the gastrointestinal mucosa (Malaisé et al., 2020). They resist the damage of pathogenic antigen attack by humoural immunity and cellular immunity and are one of the main sites of the body’s immune defence actions. Secretory immunoglobulin (S-IgA) is synthesized and secreted by plasma cells in the intestinal mucosa and is the most secreted immunoglobulin in the body. S-IgA can prevent or weaken the invasion and colonization abilities of antigenic substances and can also promote phagocytosis of antigens by phagocytes (Liu et al., 2015). The body’s immune defence process is inseparable from the existence of such immune substances. TCM can improve the body’s immunity to resist the invasion of external germs and can also fight intestinal inflammation by regulating the levels of pro-inflammatory and anti-inflammatory factors in the body. Research confirmed that Hetiao Jianpi Decoction can reduce plasma DAO and lactic acid levels and increase SIgA levels in antibiotic-associated diarrhea (AAD) rats to repair intestinal mucosal permeability and immune function (Li et al., 2020). Clinical studies have shown that GQD can reduce mouse PD-1, increase IL-2, and restore T cell function (Lv et al., 2019), thereby exerting immune function. In a study of the effect of Astragalus polysaccharide (APS) on mucosal immunity, it was confirmed that TCM can improve the overall immunity level of mice at the nonspecific immunity, humoural immunity, cellular immunity, and mucosal immunity levels (Xia et al., 2011). Another study showed that TCM can stimulate the body’s immunity by regulating intestinal flora. The Sonnenberg team in the United States performed faecal bacterial transplantation in mice to observe the relationship between ILC3 cells and the body’s immunity, and their results confirmed that ILC3 cells interact directly with TH17 to promote the production of TH1 cells and CD8+ T cells, and this interaction occurs under the action of specific intestinal microorganisms that exert intestinal-specific immune functions to resist tumourigenesis (Goc et al., 2021).

**Figure 4:**
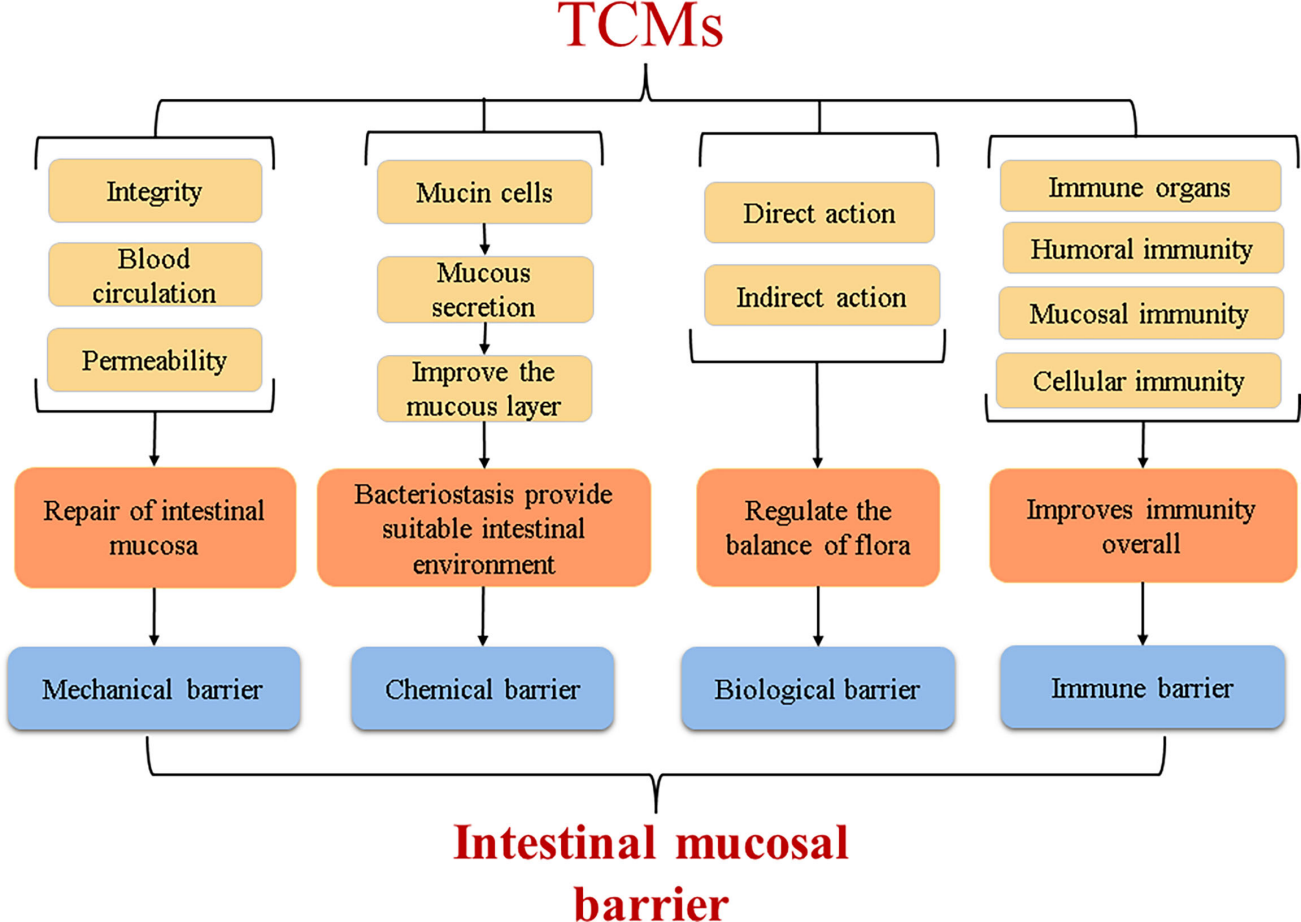
The effect of TCMs on the intestinal mucosal barrier.

**Figure 5:**
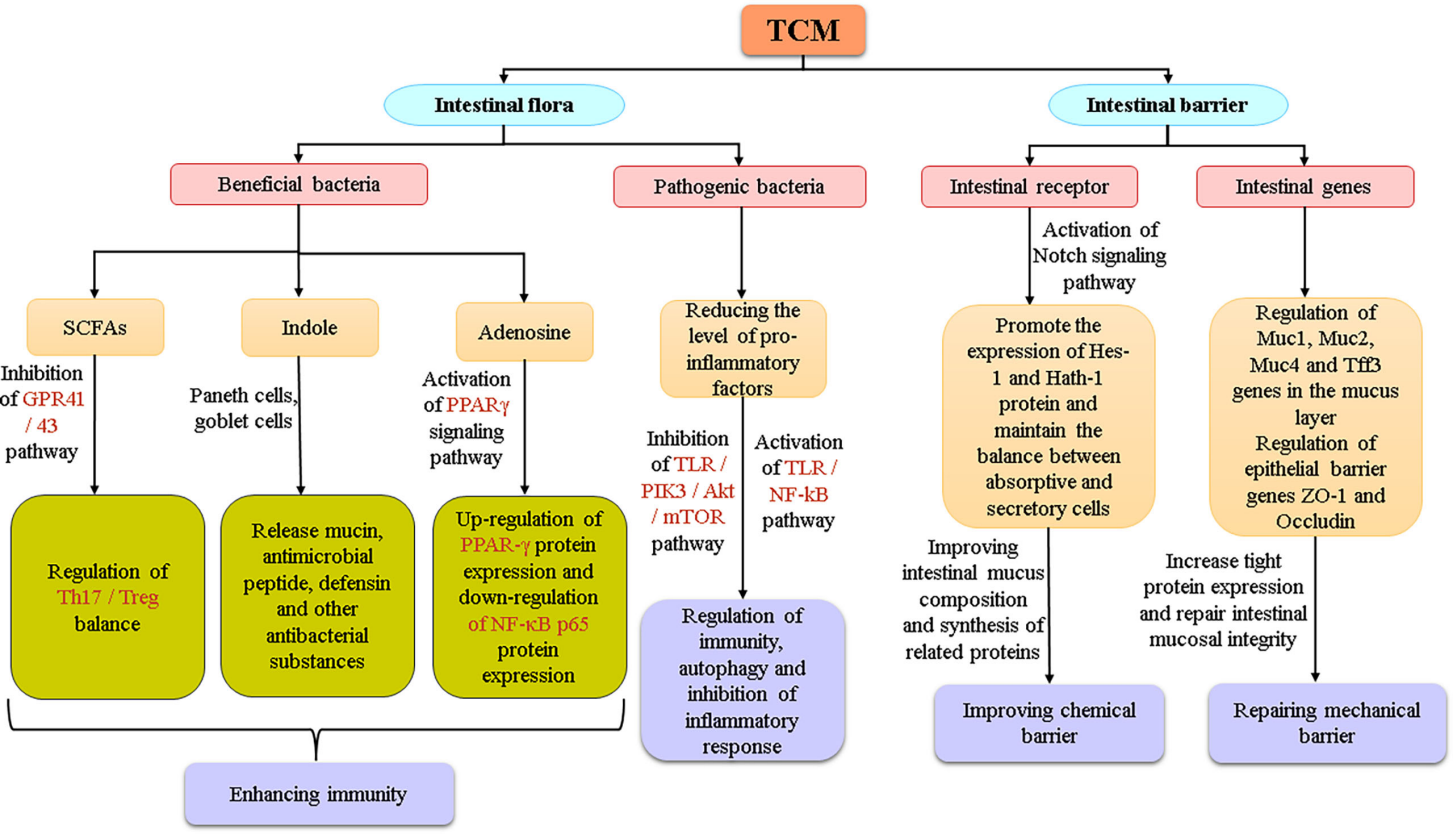
The relationship between intestinal flora and the intestinal barrier system under the action of TCM.

In the theory of Chinese medicine, the TCM could be used as a trigger or an enhancer to start the immune vitality of organs such as the spleen, and improves the body’s immune defence ability. Regarding the four layers of the intestinal mucosal barrier (**Figure 4**), basically maintain integrity of the intestinal mucosa, block the invasion of the harmful material. Studies have shown that Shaoyao Decoction can repair the intestinal mucosal barrier by regulating the expression of the Muc1, Muc2, Muc4, and Tff3 genes in the mucus layer and the epithelial barrier genes ZO-1 and Occludin. Shaoyao Decoction can also reduce the levels of proinflammatory cytokines, improve the anti-inflammatory ability of colon tissue, and increase the secretion of mucus to repair the mucosal epithelium (Chi et al., 2021). Many research results have supported that TCMs can direct effects on the intestinal tract, activate the expression of related genes and signaling pathways, or repair intestinal mucosa by regulating microbial metabolites (**Figure 5**). But it is unknown whether the intestinal parts other than the intestinal flora are related to TCM. From the perspective of intestinal absorption, the effect of intestinal tract on TCM is likely to be reflected by affecting the absorption of medicinal ingredients.

### Toxicological Effects of TCM on Intestinal Barrier and the Relationship Between Environment and Health

Most TCMs have a good repair effect on intestinal mucosal barrier and intestinal flora, but some TCMs have also brought certain damage to the intestine while achieving the effect of treating certain diseases. It has been confirmed that soya-saponins can reduce the mucosal fold height, induce the proliferation and apoptosis of intestinal cells, increase epithelial permeability, destroy intracellular connections and damage intestinal antioxidant system, eventually lead to intestinal mucosal damage (Gu et al., 2018). Soybean lectin (SBA) can bind to small intestinal epithelial cells, change the glycan structure of small intestinal mucosa, and further change the attachment sites of certain bacteria on the intestinal surface, thereby selectively stimulating the instantaneous growth of certain bacteria. On another level, SBA induction provides bacteria with rich nutrients (e.g. loss of serum proteins and increased intestinal cell loss). Besides, SBA can also destroy the intestinal mucosal immune system and reduce the secretion of immunoglobulin A (IgA), thereby inhibiting bacterial proliferation and aggravating the deterioration of intestinal mucosa (Pan et al., 2018). In addition, the planting environment of TCM has a certain influence on the effect of intestinal therapy. Toxicity of TCMs is caused not only by toxic medicinal compounds, but also by pollutants such as pesticides, herbicides and heavy metals, which can adversely affect the intestines through the human body (Feng et al., 2021). For example, aluminum accumulated in plants can promote the apoptosis of intestinal epithelial cells, destroy the structure of tight junction proteins, increase intestinal permeability;induce the activation of immune cells to secrete inflammatory factors and trigger immune response;regulate intestinal composition and enzyme activity; induce the imbalance of intestinal flora, inhibit the growth of beneficial bacteria, promote the proliferation of harmful bacteria, and then damage the four barrier of intestinal mucosa in an all-round way (Hao et al., 2022). The environment is closely related to human health. In the great cycle of nature, we should also deal with the relationship between nature and human beings, so as to achieve a win-win situation between environment and health.

## Application of TCM Research Technology in Intestinal Barrier and Intestinal Flora

The material basis of TCM for disease prevention and treatment comes from biological active parts or active chemical components, but the intestinal absorption, metabolism and excretion of drugs by biological organism is an extremely complex process. TCM is expected to develop into nano drug delivery system through careful design of nanotechnology, which comprehensively improve the medicinal value of TCM for curing and preventing diseases (Zheng et al., 2021). Nano traditional Chinese medicine (Nano TCM) refers to the effective components, original drugs and compound preparations of TCM made by nanotechnology with diameter less than 100 nm. Nano TCM includes many techniques, such as nano carrier, solid dispersion and so on. It is not only used to crush the drug to nanometer level, but also to process the prescription composition of the effective part or active component of the drug through nanotechnology, giving TCM new functions. Although nanotechnology is widely used and gradually improved, the nanotechnology of TCM is still in its infancy, and its development space needs to be excavated.

Mass spectrometry imaging technology is a high-throughput method to detect and image the metabolic changes of various components of TCMs. As a new analytical imaging technology, this method is fast and sensitive which does not require complex TCM extraction and separation. In addition, this technology can also be used to further focus on potential biomarkers, which has laid a certain foundation for the study of the interaction mechanism between TCMs and intestinal flora, and has unique characteristics in metabolic analysis, quality control and mechanism of action exploration. Advantages to help establish quality standards, explore the safety and toxicology of TCMs (Jiang et al., 2022).

Furthermore, the combination of molecular docking and network pharmacology has greatly promoted the prediction of bioactive components and shed a light on the mechanism study of TCM affecting on the intestine. Molecular docking is a computer technology based on structural design, while network pharmacology has established a powerful and comprehensive database to understand the relationship between TCM and disease networks. The combination of this two provides a theoretical basis and technical support for the construction of modern TCM based on component compatibility, and also provides a new way for the exploration of the repair mechanism of TCM on the intestinal barrier and intestinal flora (Jiao et al., 2021).

In addition, a newly developed technology can also identify the effect parts of TCM. Plant metabolomics, which could explore the interactions between plant metabolites by addressing key network components among plant small molecules, has made significant contributions to understanding the relationship between genotype and metabolic output (Hong et al., 2016). It can characterize the dynamic changes of plant metabolites and has the ability of holistic analysis, which conforms to the holistic view theory of TCM and can reflect the holistic effect of exogenous substances on organisms. This approach is good suitable for analyzing complex systems such as TCM, and is conducive to the exploration of various factors in intestinal repair (Zhang et al., 2019).

At present, the research on the mechanism of intestinal repair by TCM remains on the surface. However, based on the continuous improvement of science and technology, it is gradually systematic to explore the pathways, targets and repair mechanisms of TCM in the treatment of intestinal tract. With the continuous development of technology and the continuous improvement of scientific level, the specific mechanism behind it will eventually be clearly explored.

## Conclusions and Perspectives

TCM has a plentiful supply of natural biological components, which can comprehensively regulate the organs of the body, strengthen the spleen and lungs, regulate yin and yang, and have a remarkable effect on the defense and treatment of clinical diseases such as neurology and metabolic disorders. According to studies, 90% of the human body’s diseases are related to the intestine, and intestinal regulation is increasingly recognized as the foothold and breakthrough point of diseases. From the perspective of TCM, people’s various organs are not independent but are all interconnected and together influence the whole-body physiology. Therefore, TCMs regulate intestinal function through very complex mechanisms. Although the intestinal mucosal repair mechanisms of TCMs have not been completely and systematically detailed, their repair effect is obvious by now. There is also nanoimaging technology that can be used to track TCMs in the intestine, another avenue researchers are using to discover their mechanisms of action. We believe that the comprehensive regulatory mechanisms of TCMs in intestinal health will be gradually revealed in the future.

## Author Contributions

D-LX conceived, supervised and writing-reviewed the manuscript, QC and TL originally wrote and writing-reviewed the draft, JS and YH cofounded and co-administrated the project. All authors approved the final version.

## Funding

This research was supported financially by the National Natural Science Foundation of China (31560079, 31960074), the Science and Technology Department Foundation of Guizhou Province of China (No.[2017]5733-050, [2019]-027, [2019]5657), the Special Joint Bidding Project of Zunyi Sci & Tech Bureau and Zunyi Medical University (ZSKHHZ-2020-91) and Honghuagang Sci & Tech Project of Zunyi City (ZHKHNZT [2020]04).

## Conflict of Interest

The authors declare that the research was conducted in the absence of any commercial or financial relationships that could be construed as a potential conflict of interest.

## Publisher’s Note

All claims expressed in this article are solely those of the authors and do not necessarily represent those of their affiliated organizations, or those of the publisher, the editors and the reviewers. Any product that may be evaluated in this article, or claim that may be made by its manufacturer, is not guaranteed or endorsed by the publisher.
